# Contribution of clinical breast exam to cancer detection in women participating in a modern screening program

**DOI:** 10.1186/s12905-021-01507-x

**Published:** 2021-10-19

**Authors:** Tehillah S. Menes, Dan Coster, Daniel Coster, Shani Shenhar-Tsarfaty

**Affiliations:** 1 Department of Surgery, Tel Aviv Sourasky Medical Center, Tel Aviv, Israel; 2 Sackler School of Medicine, Tel Aviv University, Tel Aviv, Israel; 3 Blavatnik School of Computer Science, Tel-Aviv University, Tel Aviv, Israel; 4 Joyce and Irving Goldman Medical School, Faculty of Health Sciences, Ben-Gurion University of the Negev, Beer Sheva, Israel; 5 Department of Internal Medicine “C”, “D” and “E”, Tel-Aviv Sourasky Medical Center, Tel Aviv, Israel; 6 Present Address: Department of Surgery C and Surgical Oncology, Chaim Sheba Medical Center, Ramat Gan, Israel

**Keywords:** Breast cancer, Screening, Clinical breast exam, Mammography

## Abstract

**Purpose:**

Despite the controversy surrounding the role of clinical breast exam (CBE) in modern breast cancer screening, it is widely practiced. We examined the contribution of CBE in women undergoing routine screening mammography and in women under the screening age.

**Methods:**

A retrospective cohort study including all women participating in a voluntary health screening program between 2007 and 2016. All participants undergo CBE; Screening mammography is done selectively based on age, breast imaging history and insurance coverage. Data collected included demographics, risk factors, previous imaging, and findings on CBE and mammography. Cancer detection rates within 3 months of the visit were calculated separately for women undergoing routine screening mammography, and women under the screening age.

**Results:**

There were 14,857 CBE completed in 8378; women; 7% were abnormal. Within 3 months of the visit, 35 breast cancers (2.4 per 1000 visits) were diagnosed. In women within the screening age who completed a mammogram less than one year prior to the visit (N = 1898), 4 cancers (2.1 cancers per 1000 visits) were diagnosed. Only one was diagnosed in a woman with an abnormal CBE, suggesting that the cancer detection rate of CBE in women undergoing regular screening is very low (0.5 per 1000 visits). In women under the screening age (45), 3 cancers (0.4 per 1000 visits) were diagnosed; all were visualized on mammography, one had an abnormal CBE.

**Conclusions:**

The contribution of CBE to cancer detection in women undergoing routine screening and in women under the screening age is rare.

**Supplementary Information:**

The online version contains supplementary material available at 10.1186/s12905-021-01507-x.

## Introduction

As the controversy continues regarding the role of breast cancer screening [1], the American Cancer Society (ACS) updated its guidelines in 2015 for average-risk women [2]. These guidelines recommend that average-risk women start screening mammography at age 45. The ACS does not recommend clinical breast examination (CBE) for breast cancer screening in average-risk women at any age. This recommendation is based on lack of evidence of any benefit for CBE either as a stand-alone tool or in conjunction with screening mammography [2]. In contrast with the ACS guidelines, the National Cancer Comprehensive Network (NCCN) continues to recommend CBE from the age of 25 as part of the clinical encounter [3]. With the increase in sensitivity of modern mammography, the contribution of CBE is expected to decrease. In younger women, that are not included in screening programs, CBE may contribute to early diagnosis of breast cancer [4, 5].

In Israel breast cancer is the most common malignant disease. Approximately a quarter of all newly diagnosed breast cancer patients are under age 50 and almost 15% are under 45 [6] The national breast cancer screening program regularly invites all women from age 50 to 74 for a biennial mammography. The Health Ministry recommends as of February 2019 to include in the program also women between ages 45 and 49 wishing to undergo screening. The screening program was implemented in the 1990's and at present attendance rates are estimated at 75% of the population [7]. The Center for Screening and Preventive Medicine in the Tel Aviv Sourasky Medical Center provides medical screening services in addition to those provided by the health maintenance organizations. Most attendees receive these services as a benefit provided by their employer. During the visit, all women undergo different health exams including a CBE. Screening mammography is done selectively based on age, recent breast imaging history as well as their specific insurance coverage. We used this unique setup to assess the contribution of CBE to the diagnosis of breast cancer in women undergoing regular screening mammography and in women under the screening age (under 45).

## Methods

The Center for Screening and Preventive Medicine in the Tel Aviv Sourasky Medical Center provides medical screening services to adult men and women. The program is voluntary and provides general health screening services. Most participants receive the service as an employer-provided benefit. During the visit they undergo multiple screening exams including a complete physical exam, gynecological exam and different tests. Breast cancer screening includes a CBE by a surgeon (either general or breast surgeon on a rotating basis, with a wide range of experience). If the CBE is normal, based on the woman's age, recent imaging history and insurance plan, a screening mammography is performed as well, usually during the same visit. The program has no upper age limit for screening mammography. Women with abnormal CBE are recommended for further work-up with imaging based on their age: women under 30 are recommended to undergo a breast ultrasound; women aged 30 and up are recommended to first undergo a bilateral mammogram with further work-up as needed. Further testing with ultrasound and magnetic resonance imaging (MRI) is selectively recommended per treating surgeon, based on the CBE and risk assessment. In order to expedite the screening process, the tests are performed in parallel with some women being first examined by the surgeon and some undergoing imaging first. Institutional Review Board was obtained and informed consent was waived.

All women undergoing CBE with or without screening mammography in our center between December 2007 and October 2016 were included. Women with a personal history of cancer (except for skin cancer) or known BRCA pathogenic mutation were excluded, as were medical tourists and men. We excluded women with suspicious breast complaints (detailed in Additional file 1: Appendix 1). Women with non-specific complaints (Additional file 1: Appendix 1) were not excluded.

We collected data including demographics (date of birth, health and screening history), and breast cancer risk factors (family history, parity, menopausal status, etc.) Data on mammograms done before the visit was routinely obtained from the women at the time of the visit. In cases in which this data was not available the chart was reviewed and data on previous visits to the screening center with breast imaging was retrieved.

Findings on CBE, and on mammography and breast ultrasound when performed were extracted. The CBE comprised a free-text written by a physician and/or four binary values that represent the exam findings in the breast and axilla (i.e., normal or abnormal exam in each breast and axilla). We considered the CBE result positive if one of the binary values was positive. When no values were reported, a breast surgeon manually reviewed the physician's text and decided if there was a pathological finding. A positive CBE was considered when the description included one of the words detailed in the appendix (Additional file 1: Appendix 1).

Before 2015, mammography was performed using Hologic Selenia digital mammography system (Bedford, USA). Standard four-view mammographic examinations are obtained. Additional views are performed as required, breast ultrasound is recommended in all cases with dense breast. Tomosynthesis (Hologic Selenia Dimensions) was introduced in 2015 to our breast imaging center. Single reading of mammograms by one of several dedicated breast radiologists was completed without the routine use of computer aided detection. Mammograms completed within 60 days of the CBE visit were considered to be associated with the visit. The result of the mammography test was provided in free text written by the physician and transformed into binary labels (normal/abnormal) by natural language processing. A script was created in order to extract the result and recommendation from the mammography reading using a rule-based algorithm. A mammogram was considered abnormal if the Breast Imaging-Reporting and Data System (BIRADS) classification was 0, 3, 4, or 5, or if the text included a recommendation for further work up. When the ultrasound was completed together with the screening mammogram, and the imaging was reported as normal, this was considered a normal exam. A list of terms and phrases was compiled and used to create a pattern detection script. It included an action verb or noun followed by a recommended test (detailed in Additional file 1: Appendix 1). Variations of the terms were used in order to include all possible synonyms and inflections. When the pattern was identified by the script, the study was considered abnormal, otherwise it was considered to be normal. The accuracy of the script was manually reviewed and more action verbs and recommendations were added. False positives were identified and addressed. Finally, we randomly sampled 100 cases and manually reviewed the cases to confirm the efficacy of the pattern recognition script. The analysis was performed using Python programming language (version 3.5) and packages NumPy, SciPy and scikit-learn.

The outcome was defined as diagnosis of breast cancer within 3 months of the most recent CBE visit, and was ascertained by linking the women's records to the Israel National Cancer Registry (INCR). The INCR collects data on all incident cancers since 1982; completeness of data collection on solid tumors is estimated at 95–96% [8]. In order to assure complete catchment of all cancers diagnosed within 3 months of the visit, the data included visits of women up to October 1st 2016.

### Analyses

Descriptive statistics of the study population included: age, breast cancer risk factors, and number of visits per woman. The findings on CBE and mammography were summarized according to age group (under 45, 45 years and up). The groups were compared using chi-square test for categorical data, all tests were 2-sided and significance was set at 0.05.

Cancer detection rates within 3 months from the most recent CBE visit were calculated. These rates were calculated separately for the whole cohort; for different age groups; for women undergoing regular screening mammography (defined as a CBE visit less than one year from last mammogram) and for women under the screening age (under 45). These cancer cases were further scrutinized to assess the modality by which the cancer was diagnosed (CBE or imaging).

Positive predictive value (PPV) and negative predictive value (NPV) were calculated using the percentage of women diagnosed with breast cancer within 3 months of the visit. Rate precision was determined with 95% confidence intervals (CI), which were derived by using the Wilson score interval for binomial parameters.

## Results

**Fig. 1:**
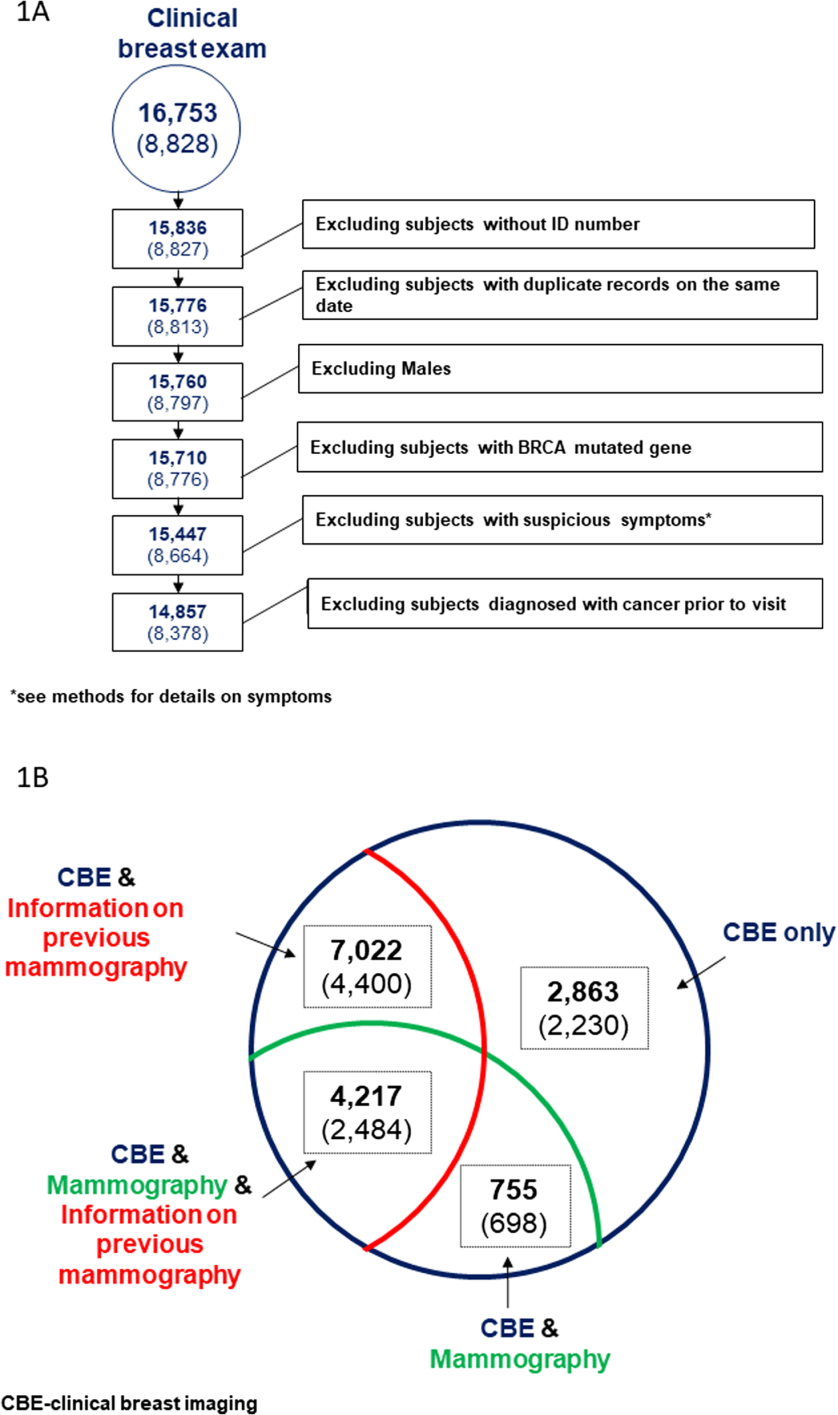
**a** Study flow chart; Summary of number of visits to the screening center during the study years, and the number of visits excluded for different reasons. Numbers in parenthesis are the actual number of women (some women had multiple visits over the years). **b** Final group of visits included in the analysis, all had a clinical breast exam (CBE) at the time of the visit. Numbers in parenthesis are the actual number of women. See Additional file 1: Appendix for details on breast symptoms

During the study years there were 16,753 screening visits with a CBE documented. After exclusion of cases that did not fill the inclusion criteria (no ID, duplicate visit entries, males, known BRCA mutation, previous history of cancer or suspicious breast symptoms), 14,857 CBE visits completed by 8378 women were included in the study (Fig. 1). Most women (5035, 60%) had only one visit. The characteristics of the study population are summarized in Table 1.

**Table 1:** Characteristics of the study population

Age group^1^	N = 14,857	(%)
< 45	6764	45.5
45 +	8093	54.5
Number of visits to screening center	N = 8378	
1	5035	60.1
2	1767	21.1
3	820	9.8
4+	756	9
Marital status^2^	N = 8378	70.4
Married	5899	15.6
Single	1308	11.5
Divorced	963	2.4
N/A	208	
Number of pregnancies^2^	N = 8378	
0	227	2.7
1	646	7.7
2	1571	18.6
3	2034	24.3
4+	2517	30.0
N/A^**3**^	1383	16.5
Number of children^2^	N = 8378	
0	262	3.1
1	864	10.3
2	2405	28.7
3	2724	32.5
4+	829	9.9
N/A	1294	15.5
Breast feeding	N = 14,857	
No	4547	30.1
Yes	7991	53.8
N/A	2319	15.6
Post-menopausal^2^	N = 8378	
Yes	2406	28.7
No	5531	66.0
N/A	441	5.3
Hormonal treatment	N = 14,857	
No	9772	65.8
Yes	2766	18.6
N/A	2319	15.6
Family history^2^	N = 8378	
No	7442	88.8
Yes	906	10.8
N/A	30	0.4
Breast complaints^4^	N = 14,857	
No	10,616	71.5
Yes	1214	8.2
N/A	3027	20.3
Previous mammogram	N = 14,857	
No	2046	13.8
Yes	9193	61.9
N/A	3618	24.3

Some of the parameters were not available for all the patients

^1^Age at the visit

^2^Information for the first visit of each participant

^3^N/A—not available

^4^Some non-specific complaints (such as bilateral pain, see methods section for details; Additional file 1: Appendix 1) were not considered abnormal and these visits were not excluded

Findings on CBE and mammography are summarized in Table 2. Seven percent of the women in both groups had an abnormal CBE (466; in women under 45, and 527 in the older group). A mass or fullness were described in 405 (41%); lymphadenopathy in 47 (5%); less common abnormalities included skin changes, nipple retraction, and nipple discharge (N = 38, 4%). In the remainder 503 (51%) the details of the abnormal exam were missing. In a third of the visits (N = 4972) a mammogram was completed as well. This proportion increased with age. In 23% (N = 1535) of visits of women under 45 a mammogram was done compared to 43% (N = 3437) of visits of women age 45 and up (*p* < 0.00001). The mammogram was coded as abnormal in 2139 (43%) of the cases. Most (N = 1799, 84%) of the exams that were coded as abnormal, were recommended for US, the majority because of increased density. A mass was noted in 185 (3.7% of all mammograms); microcalcifications in 50 (1%); and an asymmetric density in 43 (0.9%). Less common findings included lymphadenopathy, radial scar and nipple retraction (N = 11, 0.2%). In another 26 (0.5%) exams that were recommended for biopsy details were missing. Overall 2% of the women undergoing mammography were recommended to undergo a biopsy.

**Table 2:** Findings on CBE and mammography according to age groups and cancer detection rates within 3 months

	< 45 years		45 years and up	
	N (%)	Cancer^1^, N (per 1000 exams)	N (%)	Cancer^1^, N (per 1000 exams)
*CBE*^*2*^				
Normal	6298 (93)	2 (0.3)	7566 (94)	22 (2.9)
Abnormal	466 (7)	1 (2.1)	527 (7)	10 (19)
*Mammogram*				
Normal	655 (43)	0	2178 (63)	1 (0.5)
Abnormal	880 (57)	3 (3.4)	1259 (37)	27 (21.5)

^1^within 3 months of visit, ^2^CBE-clinical breast exam

Within 3 months from the most recent CBE visit, 35 breast cancers were diagnosed (2.4 per 1000 visits). Three were in 6764 women under age 45 (0.4 per 1000 visits), 5 cancers were diagnosed in 2440 women aged 45–49 (2.1 per 1000 visits); 12 in 3773 women aged 50–59 (3.2 per 1000 visits) and 15 in 1880 women 60 year and above (8 per 1000 visits).

Thirty-two cancers were diagnosed in women 45 years and older (4 cancers per 1000 visits, Table 2). In 10 cases the CBE was abnormal. However, the cancer was incidental to the clinical finding in 4; in one case it was difficult to correlate the clinical finding with the location of the cancer. In most (27; 96%) women diagnosed with cancer who completed a mammogram during the visit, it was abnormal.

In the group of women considered to be within the screening period (reporting a mammogram done less than 1 year prior to the visit, N = 1898), 4 cancers were diagnosed within 3 months of the visit; only one of which was in a woman with an abnormal CBE and mammogram; suggesting that in women undergoing yearly screening mammograms CBE can detect an additional 0.5 cancers per 1000 exams. One of these women had a normal CBE and mammogram suggesting that this cancer was missed during the visit.

In the group of women under 45 (N = 6764) 3 cancers were diagnosed. In one woman, this was secondary to a palpable breast mass which was subsequently visualized on mammography. This woman had no family history or known risk factors for breast cancer. In the two other women, the cancer was diagnosed on mammography whereas the CBE was normal.

Ten (29%) of all the cancers were diagnosed at stage 0 (in situ); 15 (43%) at stage 1, and 4 (11%) at stage 3. There was no information on the stage in the remainder 6 (17%).

The positive predictive value (PPV) of an abnormal CBE varied according to the women's age. In women under 45 the PPV for an abnormal CBE was 2 per 1000 whereas in women 45 and up, the PPV was 19 per 1000 abnormal exams. However, these values include cases in which the palpable abnormality was incidental to the cancer. The NPV for CBE in both age groups was close to 100%.

## Discussion

We report here the findings on CBEs done as part of health screening tests in women participating in a modern screening program. During the study period 14,857 CBEs were completed. Thirty-five (0.2% of 14,857 CBE visits) women were diagnosed with breast cancer within 3 months of the visit; most (N = 30) of these cancers were visualized on mammography completed during the visit. The detection of cancer by CBE alone in women with a recent mammogram was rare; of 1898 women with a mammogram done within one year of the visit, only one woman diagnosed with cancer had an abnormal CBE, her mammogram was abnormal as well. In women under 45, three cancers were diagnosed after 6764 CBEs. Only one of these cancers was identified on CBE, all were visualized on mammography.

Several randomized studies evaluated the role of CBE in countries where screening mammography is not prevalent [9–11]; some showing an associated shift to earlier stage at diagnosis. Data on the contribution of CBE to cancer detection in women participating in a screening mammography program and in women under the age of screening is lacking. No randomized studies compared the combination of mammography and CBE to mammography alone. The contribution of CBE to detection of breast cancer can be assessed indirectly using data from early screening trials that compared no screening to screening with mammography and CBE. In these early trials when mammography was less sensitive, the proportion of cancers diagnosed by CBE alone ranged widely between 3 and 45% [12–16]. Despite lack of data, CBE was incorporated into many screening programs. Based on data from these observational studies, the proportion of cancers detected by CBE alone ranges between 0 and 31% [17].

The Canadian screening programs in Ontario included centers that offered CBE with mammography and centers that offered mammography alone. Based on data from these programs, an additional 0.3–0.4 cancers were detected per 1000 women screened with CBE [18, 19]. In a study examining the sensitivity of CBE in asymptomatic women that were diagnosed and died of breast cancer, CBE identified the tumor in only 21% [20].

CBE may contribute differentially in subgroups of women. Increased cancer detection was reported in older low-risk women receiving hormone therapy and in women with dense breasts [21, 22].

These reports are based on studies that used older mammography technology, and without the addition of ultrasound that is recommended in women with dense breasts, and therefore probably overestimate the contemporary contribution of CBE in women undergoing regular screening.

Adding CBE to a screening program is not without a price. In the Canadian centers the supplementation of CBE to mammography was associated with an increase in the abnormal call back rates and false-positive rates. For each additional cancer detected by CBE per 10,000 women screened, there were an additional 55 false-positive screens [19]. We found that regardless of age, 7% of women undergoing CBE had an abnormal exam. The potential harms of CBE must be taken into consideration together with the added costs of the exam, and associated anxiety when weighing the small contribution of CBE to detection of cancer.

Our study has several limitations; assessment of time elapsed from previous mammogram was for the most, based on self-report and therefore subject to recall bias. Findings on CBE can be equivocal and subject to interpretation. We used a broad definition for abnormal mammogram, including all exams that recommended completion of breast ultrasound. We considered cancers detected within 3 months after the visit to be associated with the visit. This definition may miss cancers that were diagnosed subsequently (if the patient did not complete work-up of a clinical or imaging finding within 3 months of the visit), and include cancers that were diagnosed incidental to the visit. CBE may be more effective in certain high-risk groups, such as carriers of pathogenic mutations in BRCA or women with a family history of breast cancer; our cohort was not large enough to perform subgroup analyses. Women participating in the screening program may not represent the population; this may affect the ability to generalize our results to other populations.

Our results should be interpreted with caution. We assessed the contribution of CBE in asymptomatic women. Despite universal screening programs, many cancers are self-diagnosed or symptomatic; some are not apparent on routine mammography. One cannot over-emphasize the importance of prompt evaluation of breast symptoms even when imaging is interpreted as normal. Furthermore, a visit with a breast specialist may benefit the woman regardless of the CBE if it used to raise breast health awareness, assess her personal risk and recommend appropriate surveillance.

## Conclusions

In summary, in this study examining the contribution of CBE to cancer diagnosis in women participating in a modern breast screening program, CBE was associated with a high proportion of abnormal findings (regardless of age), and its contribution to early diagnosis of cancer was rare in all age groups. With the adoption of more personalized breast cancer screening in the future, the role of CBE in specific subgroups of women will need to be evaluated.

## Supplementary Information

**Additional file 1:** Appendix 1 - Dictionary of words used to identify abnormal symptoms, non-specific symptoms and abnormal CBE and mammogram.

## Data Availability

The datasets generated and/or analyzed during the current study are not publicly available due ethical/privacy reasons. Upon request the analyses generated from the data are available from the corresponding author.
