# Supplementary material for: Contribution of clinical breast exam to cancer detection in women participating in a modern screening program
Source: BMC Womens Health. 2021 Oct 19;21:368. doi: 10.1186/s12905-021-01507-x (PMC8524962; doi:10.1186/s12905-021-01507-x)
Supplement: Supplementary file 1 — Additional file 1: Appendix 1 - Dictionary of words used to identify abnormal symptoms, non-specific symptoms and abnormal CBE and mammogram. [file 12905_2021_1507_MOESM1_ESM.docx]

Appendix 1.

**Methods:**

**Dictionary of words used to identify women with suspicious symptoms:**

- lump; mass; bulge; fullness; lumpy; thickening; finding; lesion; uneven; swelling; congestion; change in size; new asymmetry; enlargement; itching; discomfort; burning; discharge; secretion; inflammation; pus

**Dictionary of words used to identify women with non-specific symptoms:**

- breast pain; sensitivity; tingling; stabbing; sensation;

**Dictionary of words used to identify abnormal clinical breast exam (CBE):**

Skin changes: inflammation; erythema; hematoma;

Findings on inspection:

- nipple retraction;
- secretion/discharge;

Palpable findings:

- Breast: mass; nodule; nodular; nodularity; ridge; lesion; finding; fullness; swelling; induration; dense tissue; thickening; bulge; fibrotic consistency;
- Axilla: lymphadenopathy; palpable axillary lymph node;

Any finding which was localized or asymmetric to the other breast:

- marked asymmetric fibrocystic changes;
- asymmetric glandular tissue;

Equivocal findings were also considered abnormal: questionable fullness

**Abnormal mammograms:**

Mammograms were identified as abnormal using a pattern detection script:

All phrases after an action verb or noun followed by a word from a dictionary of follow-up tests/exams were extracted and used to identify abnormal results.

Action words or verb dictionary:

- required; to complete; completion; recommend; recommended; needed; completion; work-up; done

Test/exam dictionary:

- ultrasound; sonogram; US; sonography; MRI; needle; biopsy; trucut; vacuum; mammotome;
